# Sex Hormones Modulate the Relationship Between Global Advantage, Lateralization, and Interhemispheric Connectivity in a Navon Paradigm

**DOI:** 10.1089/brain.2017.0504

**Published:** 2018-03-01

**Authors:** Belinda Pletzer, TiAnni Harris

**Affiliations:** Department of Psychology, Centre for Cognitive Neuroscience, University of Salzburg, Salzburg, Austria.

**Keywords:** sex differences, attention, lateralization, interhemispheric connectivity, global advantage, Navon paradigm

## Abstract

Sex, stimulus material, and attention condition have previously been related to global advantage (GA; faster responses to global targets than to local targets) on the one hand and lateralization during global–local processing on the other hand. It is presumed that the lateralization of brain functions is either related to the inhibitory influence of the dominant on the nondominant hemisphere or reduced excitation between hemispheres. However, a direct relationship between the GA and lateralization and interhemispheric connectivity has not been previously established. In this study, 58 participants (29 men, 29 naturally cycling women) completed a Navon paradigm, modulating attention condition (divided vs. focused) and stimulus material (letters vs. shapes) during functional magnetic resonance imaging. The size of the GA effect, lateralization indices, interhemispheric connectivity, and sex hormone levels were assessed. In summary, this study suggests that interhemispheric connectivity during global–local processing is affected by sex and material. Furthermore, the relationship between interhemispheric connectivity, lateralization, and behavior was modulated by sex and sex hormones. Results suggest (1) differential roles of interhemispheric connectivity for lateralization in men and women and (2) differential roles of lateralization for behavior in men and women. Importantly, the classic assumption that a more negative connectivity leads to stronger lateralization, which in turn leads to a stronger GA effect, was observed in men, whereas the opposite pattern was found in women. The relationship between connectivity and lateralization was mediated through testosterone levels, whereas the relationship between lateralization and behavior was mediated through progesterone levels. Results are discussed in light of differential functions of inhibitory and excitatory interhemispheric processes in men and women.

## Introduction

Introduced by Navon (1977), global–local processing is traditionally studied using hierarchical visual stimuli, that is, global structures made up of smaller local parts. Participants are typically asked to respond to one or two targets (*target detection paradigm*) either at any level (*divided attention paradigm*) or only at the global or local level (*selective attention paradigm*). Reaction times of responses to global and local targets are used to assess the *global advantage *(GA) *effect, a* measure of global–local processing. The GA effect describes the overall tendency of recognizing global targets faster than local targets.

The GA effect was found to be modulated by interindividual factors such as sex (Pletzer, 2014; Razumnikova, 2011; Roalf et al., 2006), as well as a variety of task factors, including the spacing of local elements, timing, position, and visual angle of the stimuli (Boer and Keuss, 1982; Grice et al., 1983; see Kimchi, 1992 for a review; Martin, 1979). Task factors that are less commonly studied with regard to their relationship with the GA effect are *stimulus material* and *attention condition.*

Regarding sex differences, a stronger GA in men than in women has consistently been found with hierarchical letter stimuli (Pletzer, 2014; Razumnikova, 2011; Roalf et al., 2006), but has been controversially discussed for other stimulus materials (Kimchi et al., 2009), with some inconsistencies regarding the attention paradigm. Most recent results also suggest hormonal influences on global–local processing with sex differences in the GA effect being restricted to the luteal cycle phase in women (Pletzer, 2014).

Regarding stimulus material, Kimchi and colleagues (2009) were unable to identify differences in GA between shape and line stimuli. Pletzer (2014), however, found differences in GA between hierarchical letter and shape stimuli, with a larger GA for the letter than shape stimuli.

Concerning attention condition, Pletzer (2014) found the GA effect to be larger in a selective attention paradigm than in a divided attention paradigm. Similarly, autistic children show local precedence in a divided attention task, but global precedence in a selective attention task (Plaisted et al., 1999). Furthermore, patients with visuospatial neglect are only impaired in global processing during a divided attention paradigm but not during a selective attention paradigm (Lux et al., 2004).

The factors described to influence GA, that is, sex, stimulus material, and attention condition, have also been described to influence another important aspect of global–local processing, that is, lateralization. Hemispheric asymmetries are often seen as a result of interhemispheric interactions (Bloom and Hynd, 2005). It is, however, still a matter of debate whether the primary role of the corpus callosum is excitatory or inhibitory (Bloom and Hynd, 2005). Inhibitory models of the corpus callosum see a dominant and a nondominant hemisphere for simple tasks. For these tasks, intrahemispheric processing within the task-specific dominant hemisphere takes place, whereas the nondominant hemisphere is inhibited through corpus callosum, saving resources such as energy and time (theory of metacontrol) (see van der Knaap and van der Ham, 2011 for a review). Thus, although there is much evidence for excitatory functions of the corpus callosum, inhibitory functions seem to play a major role in the emergence of lateralization patterns and hemispheric asymmetries (*lateralization by inhibition*, Chiarello, 1995).

A variety of studies, primarily using hierarchical letter stimuli, suggests hemispheric asymmetries in global–local processing with a right hemispheric advantage for global features and a left hemispheric advantage for local features (see van Kleeck, 1989 for a meta-analysis). Evidence comes from behavioral visual hemifield studies (Hübner and Studer, 2009; Martinez et al., 1997; Peyrin et al., 2003; Yovel et al., 2001), studies conducted on a brain-damaged patient (Hellige, 1993; Lamb and Robertson, 1989; Lamb et al., 1990; Robertson et al., 1988; Robertson and Lamb, 1991), electro-encephalographic studies (Heinze et al., 1998; Johannes et al., 1996; Yamaguchi et al., 2000), and neuroimaging studies (Fink et al., 1996; Heinze et al., 1998; Martinez et al., 1997; Weissman et al., 2003, 2005).

Regarding sex differences, it has been argued that women have an advantage in cognitive functions lateralized to the left hemisphere, whereas men have an advantage in cognitive functions lateralized to the right hemisphere (Clemens et al., 2006; Moffat and Hampson, 1996; Tomasi and Volkow, 2011), which is in line with the results of stronger local processing in women, but stronger global processing in men. Furthermore, men show stronger lateralization during a variety of cognitive tasks than women (McGlone, 1980; Shaywitz et al., 1995). Also, variations of hemispheric asymmetries are more pronounced in women than in men (McGlone, 1980). Hausmann and Güntürkün (2000) argue that these variations in female participants are attributable to hormonal variations across the menstrual cycle. Specifically, hemispheric asymmetries during a variety of cognitive tasks were reduced in women in their luteal cycle phase (high estradiol and progesterone) compared with their follicular cycle phase (low estradiol and progesterone). In fact, hemispheric asymmetries in women during their follicular cycle phase were comparable with those in men. These findings have been replicated several times using different methodologies (Hausmann and Bayer, 2010 for a review; Hausmann et al., 2013 for an EEG study; Weis and Hausmann, 2010 for a functional magnetic resonance imaging [fMRI] study). However, it remains an unresolved issue, whether progesterone or estradiol matter in that respect (e.g., Hausmann and Bayer, 2010). These differences in lateralization have been linked to differences in interhemispheric connectivity between men and women (Bloom and Hynd, 2005; Weis and Hausmann, 2010).

Regarding stimulus material, Fink and colleagues (1997) reported inverted hemispheric asymmetries for hierarchical object stimuli. In a neuroimaging study, he found increased right hemispheric activation in the inferior occipital cortex for local targets and increased left hemispheric activation in the lingual gyrus for global targets with hierarchical objects. His assumption that stimulus material plays a major role for hemispheric asymmetries during global–local processing did, however, not receive much attention. A partial replication comes from a visual hemifield study in children (Keita and Bedoin, 2011), which observed a left hemispheric dominance for local letters, but right hemispheric dominance for local objects with no hemispheric asymmetries for global objects.

Regarding attention condition, hemispheric asymmetries are usually stronger in divided attention paradigm than in selective attention paradigm (see Fink et al., 1996 for an fMRI study; see Hübner et al., 2007 for a review; see Yovel et al., 2001 for an EEG study). However, interhemispheric connectivity has not been studied with respect to stimulus material and attention condition in the Navon paradigm.

Summarizing from the mentioned findings, global–local processing has been linked to hemispheric asymmetries. These asymmetries strongly depend on the attention condition, but have also been linked to stimulus material and sex, as well as sex hormones: factors that have also been linked to the GA effect. However, the role of interhemispheric interactions for the GA effect has previously not been discussed. In this study, we utilize a Navon paradigm during fMRI to tackle this question. In particular, this study seeks to investigate whether sex, material, and attention condition affect interhemispheric connectivity during global–local processing. If lateralization during global–local processing results from interhemispheric inhibition, we expect stronger interhemispheric inhibition during divided attention as opposed to selective attention. Furthermore, if the findings of increased lateralization in men also transfer to global–local processing, we expect stronger interhemispheric inhibition in men than in women. To confirm the assumption that lateralization during global–local processing results from interhemispheric inhibition, we address whether interhemispheric connectivity can be related to lateralization during global–local processing. Most importantly, we seek to investigate whether the GA effect can be predicted by either lateralization or interhemispheric connectivity. The modulatory role of the sex hormones progesterone and testosterone will be explored in that respect.

## Method

### Participants

A total of 86 healthy participants (45 men, 41 women) were recruited for this study. Exclusion criteria were physical, endocrine and/or mental illness, hormonal contraception or medication, nonremovable metal in or on the body, and left-handedness. Female participants were required to have a regular menstrual cycle between 21 and 35 days (Fehring et al., 2006) and were tested during their luteal cycle phase. Based on participants' self-reports about their onset of last period and cycle duration, ovulation was calculated 14 days before the assumed onset of their next period and confirmed by commercial ovulation test. Scanning sessions were scheduled 3–10 days after ovulation. The study complied with the ethical standards as stated in the Declaration of Helsinki and was approved by the local ethics committee. Participants also signed an informed consent, in which all requirements were listed and explained.

A total of 25 participants were excluded from analyses due to high error rates (>50%) in the Navon task (see *Navon task* section) and an additional three women had to be excluded due to a low concentration of progesterone in their luteal phase. Follow-up reports indicated that these women missed their menstruation the next month. Consequently, data were analyzed for 58 participants, 29 men (mean age 25.41, SD 4.29) and 29 women (mean age 25.41, SD 5.00). Age ranged from 18 to 40 and did not differ significantly between men and women (t_(56)_ = 0.34, *p* = 0.74). Mean cycle duration of the female participants was 29 days (SD 2.67). Mean cycle day was 21.75 (SD 3.70).

### Navon task

**FIG. 1.**
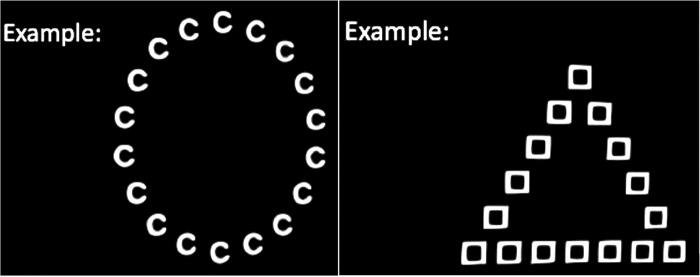
Example stimuli used during the Navon task.

For this study, a Navon paradigm using traditional hierarchical stimuli was chosen (Navon, 1977). Participants completed one session using hierarchical letter stimuli, that is, global letters consisting of local letters and one block using hierarchical shape stimuli, that is, global shapes consisting of local shapes (Fig. 1). Stimuli consisted of letters “C,” “D,” “O,” “U,” and “V” and shapes “triangle” (T), “square” (S), “circle” (C), “hexagon” (H), and “pentagon” (P). The distance between local letters/shapes was the same height as the local letters/shapes themselves (average spacing). The same letter or shape was never shown simultaneously at the global and local levels. After a fixation cross presented for 500 ms, stimulus presentation time was 150 ms, followed by an interstimulus interval of 1500 ms. All participants responded with their dominant, that is, right, hand (left button yes/right button no).

For each block, participants were randomly assigned two targets, that is, two letter targets and two shape targets. Their task was to identify the targets either irrespective of the level at which they appeared (divided attention condition) or only at an instructed level (global or local, selective attention condition). Participants responded yes (left button) if they identified a target and no (right button) otherwise. No stimulus contained targets at both the global and local levels, resulting in 18 possible combinations of letters and shapes. Thus, during each block, participants completed three runs, one for the divided attention condition and two for the selective attention condition varying the level of focus (global and local). A total of 108 (36 global, 36 local, 36 no target, and 36 null event) stimuli (each combination three times) were presented during each run.

Both, the order of materials and the order of levels in the selective attention condition, were counterbalanced. A total of 27 participants (15 men, 12 women) completed the letter condition first, 31 participants (14 men, 17 women) the shapes condition. In the selective attention condition, 29 participants (15 men, 14 women) were first instructed to only respond to global targets and then to only respond to local targets. A total of 29 participants (14 men, 15 women) were first instructed to only respond to local targets and then to only respond to global targets. Participants, who received the global instructions first in the letters task, also received the global instructions first in the shapes task. The divided attention condition was always presented before the selective attention condition to avoid priming of focus.

Stimuli were presented using Presentation Software (version 0.71, 2009; Neurobehavioral Systems, Inc., Albany, CA) on an magnetic resonance-compatible back-projection screen. For each material and attention condition, the GA effect was calculated as standardized contrast (compare Zhang, 2010), that is, the difference in mean reaction times between local and global targets divided by the pooled standard deviation, for each participant. Before the MRI session, each participant completed a training trial for the Navon task.

### fMRI data acquisition

Functional and high-resolution structural images were acquired on Siemens Magnetom TIM Trio 3 Tesla scanner (Siemens Healthcare). For functional images, we used a T2*-weighted gradient echo planar (EPI) sequence (whole brain coverage, TE = 30 ms, TR = 2250 ms, flip angle 70°, slice thickness 3.0 mm, matrix 192 × 192, FOV 192 mm, in-plane resolution 2.6 × 2.6 mm). Each run consisted of 114 scans, comprising 36 transversal slices oriented parallel to the AC-PC line acquired in descending order. Structural images were shot with a T1-weighted 3D MPRAGE sequence (192 sagittal slices, slice thickness = 1.0 mm, TE 2.9 ms, TR 2.3 s, TI delay 900 ms, FA 9.0°, FOV 256 × 240 mm).

### fMRI data analysis

SPM8 (www.fil.ion.ucl.ac.uk/spm) standard procedures and templates were used to analyze the functional images. The first six images of each session were discarded. Preprocessing took place in five steps: (1) realignment and unwarping (Andersson et al., 2001), (2) slice time correction, (3) segmentation and normalization of structural images to MNI standard stereotactic space, (4) coregistration of functional and structural images, and (5) normalization of functional images using the parameters obtained in step 3. To enhance activation detection, normalized functional images were resampled to isotropic 3 × 3 × 3 mm voxels and smoothed with a 6 mm Gaussian kernel.

A two-stage mixed effects model was applied. At first level, the parameter estimates for each subject and item category were calculated by a canonical hemodynamic response function in the context of a general linear model. Only correctly solved trials were included. For both materials and attention conditions, global targets, local targets, nontargets, and null events were modeled as separate conditions. The six movement parameters were also included as regressors in the model. A high pass filter cutoff was set at 128 sec and autocorrelation correction was performed using an AR(1) model (Friston et al., 2002). For each condition of the Navon task contrast comparing global targets with null events and local targets with null events were defined at first level.

These contrasts were entered into a flexible factorial model at second level, including level (global targets vs. local targets), attention, and material as within-subjects factors and sex as a between-subjects factor. We then defined positive and negative T-contrasts representing all main effects and interactions. The primary threshold was set to *p* < 0.001 uncorrected and family-wise error correction was applied at peak level (p_FWE_ < 0.05).

### Lateralization indices

Lateralization indices are defined as the measure of hemispheric asymmetry of activation (Wilke and Lidzba, 2007). For global and local targets, lateralization indices were calculated using the LI toolbox (Wilke and Lidzba, 2007) for SPM8 for each material and attention condition for the occipital and parietal lobes. For statistical analyses, the difference between lateralization of local targets and lateralization of global targets was calculated as a measure of differential lateralization between global and local targets. Since positive lateralization indices indicate left lateralization, a positive lateralization difference score thus indicates stronger left lateralization for local targets than for global targets.

### Connectivity analyses: psychophysiological interactions

Regions of interest (ROIs) were defined as 6 mm spheres around group average peak voxels in the left and right occipital and parietal lobe. For each individual subject, ROIs were allowed to shift to the nearest local maximum within a 6 mm radius. fMRI time series were extracted from each ROI. Using the toolbox for psychophysiological interactions (PPI), this time series was multiplied with the hemodynamic response function (HRF) convolved regressors for global targets vs. null events and local targets vs. null events to generate PPI regressors modeling increased connectivity during global or local targets, respectively. The PPI regressor, the original fMRI time series, and the HRF convolved task regressor were then entered into the first-level analysis to identify areas in which activity during global or local targets was related to activity in each ROI. A contrast over the PPI time series regressor was defined for each participant at first level and entered into a flexible factorial design at second level, including level, attention, and material as within-subjects factors, as well as sex as between-subjects factors. The primary threshold was set to *p* < 0.001, uncorrected and family-wise error correction was applied at peak level (p_FWE_ < 0.05). To relate interhemispheric connectivity to lateralization and behavior, principal eigenvalues were extracted from areas displaying significant negative interhemispheric connectivity with our ROIs.

### Hormone analysis

Before and after the experiment, saliva samples were collected. Saliva samples were stored at −20°C and centrifuged at 3000 rpm for 20 min before hormone assessment. Sex hormone levels were quantified from saliva samples using DeMediTec ELISA kits for progesterone and testosterone. As expected, testosterone levels were significantly higher in men (M = 127.51; SD = 74.97) than in women (M = 47.60; SD = 21.01) (t_(56)_ = 5.53, *p* < 0.001). Progesterone levels were significantly higher in luteal women (M = 179.53.; SD = 152.03) than in men (M = 70.03; SD = 77.62) (t_(56)_ = −3.44, *p* = 0.006).

### Statistical analyses

Statistical analyses were carried out in R 3.2.2. Linear mixed effects models were utilized using the *lmer* function of the *lme4* package. Dependent variables were the GA effect, the lateralization difference score, and interhemispheric connectivity. All models control for repeated measurement by modulating participant number as a random factor. For each dependent variable, a baseline model including only the random factor was tested in a first step, to determine whether they were significantly different from 0. In a second step, the baseline model was updated using the *update* function to include “sex,” “material,” (letters vs. shapes) and “attention condition” (divided vs. selective) as fixed effects and all possible interactions between fixed effects. Nonsignificant interactions were backward eliminated using the *step* function of the *lmerTest* package at its default settings to create minimum models, including only the factors and interactions relevant for explaining the dependent variable. The step function does approximate degrees of freedom through a Satterthwaite approximation. Results of these minimum models are reported. To evaluate hormonal influences, these final models were updated by adding progesterone and testosterone as fixed effects. To evaluate interrelations between lateralization and interhemispheric connectivity, interhemispheric connectivity was added as fixed effect to the manipulation model of the lateralization difference score. To predict the GA effect, the lateralization difference score and interhemispheric connectivity were added as fixed effects to the manipulation model. In manipulation models, all dependent and independent variables were scaled to obtain effect size estimates *b* based on standard deviations, which are similar to Cohen's d.

## Results

### Behavioral results

The GA effect describes the overall tendency for people to respond faster to global targets than to local targets. Responses to global targets were significantly faster than responses to local targets (b_GA_ = 0.53, SE = 0.04; t_(174)_ = 13.95, *p* < 0.001). To assess whether global–local processing was affected by sex, material, or attention condition, the GA effect was subjected to a linear mixed effects model (formula: GA ∼1|PNr + sex*material*attention).

**FIG. 2.**
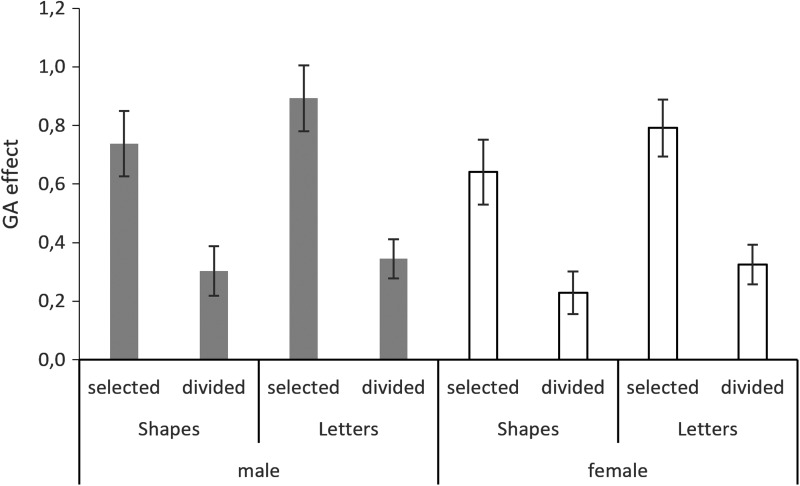
Global advantage effect. The global advantage (GA) effect was not affected by sex and only by trend stronger for letter stimuli than for shape stimuli. It was significantly stronger during the selective attention condition than during the divided attention condition.

The main effect of sex and all interactions was nonsignificant and thus removed from the model. The GA effect was by trend larger with letter stimuli than with shape stimuli (*main effect of material*: b = 0.20, SE_b_ = 0.11, t_(172)_ = 1.83, *p* = 0.07) and significantly lower during the divided attention condition than during the selective attention condition (*main effect of attention condition*: b = −0.85, SE_b_ = 0.11, t_(173)_ = −7.64, *p* < 0.001; compare Fig. 2).

To test for modulatory effects of sex hormones, testosterone and progesterone were entered as predictors in the final model (formula: GA ∼1|PNr + attention*hormone).

Testosterone was not related to the GA effect. Progesterone showed a significant negative association with the GA effect (*main effect of progesterone*: b = −0.28, SE_b_ = 0.09, t_(56)_ = −3.30, *p* = 0.002), which was significantly stronger during the selective attention condition than during the divided attention condition (attention*progesterone: b = 0.24, SE_b_ = 0.11, t_(172)_ = 2.16, *p* = 0.03). Accordingly, the GA effect was stronger the lower a participant's progesterone levels, particularly in the selective attention condition.

### Neuroimaging results

Overall, the Navon paradigm activated a bilateral parieto-occipital network with activation peaks in the left and right occipital lobe ([−45, −70, −11], [45, −67, −11]), followed by activation peaks in the left and right parietal lobe ([−42, −37, 40], [45, −37, 46]). Activation peaks of the individual conditions only deviated slightly from the overall activation peaks (compare Supplementary Table S1; Supplementary Data are available online at www.liebertpub.com/brain). Thus, these coordinates were chosen as ROI centers for connectivity analyses (see *PPI results* section). Deactivations were observed bilaterally in the inferior parietal gyri, precuneus, anterior cingulate cortex, and medial prefrontal cortex. Both activation and deactivation were stronger during the divided attention condition than during the selected attention condition. Sex and material did not influence overall activation patterns.

**FIG. 3.**
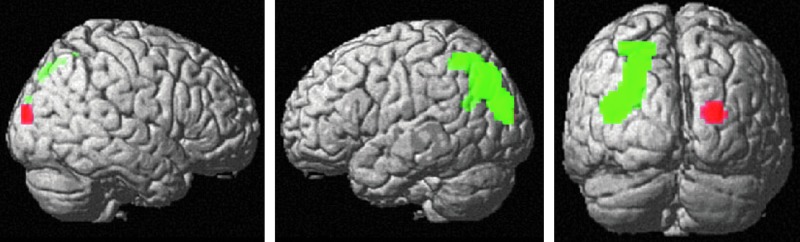
Activation differences between global and local targets. Global targets showed significantly stronger activation than local targets in the right occipital lobe (red). Local targets showed significantly stronger activation than global targets in the left occipital lobe (green).

Across conditions, global targets showed significantly stronger activation than local targets in the right occipital lobe ([24, −94, 13], T = 4.66, k = 26 voxels, p_FWE_ = 0.033), whereas local targets showed significantly stronger activation than global targets in the left occipital lobe ([24, −76, 40], T = 4.63, k = 370 voxels, p_FWE_ = 0.036) Figure 3. This effect was not modulated by sex material or attention condition.

### Lateralization indices

Lateralization indices represent the extent to which activation is stronger in one hemisphere than the other. Positive values indicate left lateralization, negative values indicate right lateralization. Overall activation in the occipital and parietal lobe was slightly left lateralized. The lateralization difference score indicates the extent to which activation was stronger left lateralized for local targets than for global targets. There was a strong association between occipital and parietal lateralization difference scores, that is, the higher the lateralization in the occipital lobe, the higher was the association in the parietal lobe (b = 0.23, SE_b_ = 0.06, t_(173)_ = 3.61, *p* < 0.001). To evaluate whether lateralization was affected by sex, material, or attention condition, the lateralization difference scores for the occipital and parietal lobes were subjected to linear mixed effects models (formula: LatDiff ∼1|PNr + sex*material*attention).

#### Occipital lobe

**FIG. 4.**
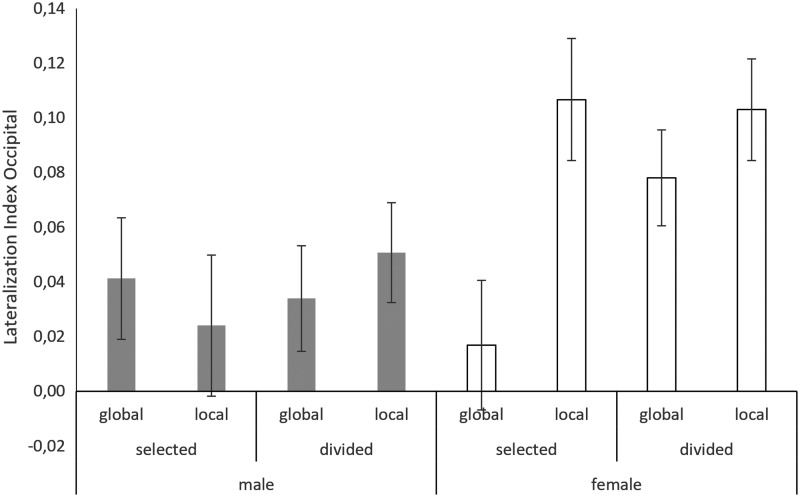
Lateralization indices in the occipital lobe. More positive values indicate stronger left lateralization. In men, lateralization indices did not differ between global and local targets. In women, local targets were significantly more left lateralized than global targets, particularly in the selective attention condition.

In the occipital lobe, the lateralization difference score was significantly larger for women than for men (*main effect of sex*: b = 0.57, SE_b_ = 0.19, t_(56)_ = 3.00, *p* = 0.004) and more so in the selective attention condition than in the divided attention condition (sex*attention: b = −0.53, SE_b_ = 0.25, t_(172)_ = −2.15, *p* = 0.03; Fig. 4). The main effect of material and its interactions were nonsignificant and thus removed from the model.

To test for modulatory effects of sex hormones, testosterone and progesterone were entered as predictors in the final model (formula: GA ∼1|PNr + sex*attention*hormone). When including testosterone in the model, the main effect of sex on the lateralization difference score in the occipital lobe disappeared. Instead, testosterone related negatively to the occipital lateralization difference score (*main effect of testosterone*: b = −0.30, SE_b_ = 0.09, t_(56)_ = −3.15, *p* = 0.003), that is, the lateralization difference between global and local targets was stronger, the lower the participant's testosterone level. This association was stronger in the selected attention condition than in the divided attention condition (testosterone*attention: b = 0.37, SE_b_ = 0.12, t_(172)_ = 3.05, *p* = 0.003). Progesterone did not modulate the effects of sex or attention on the lateralization difference score in the occipital lobe.

#### Parietal lobe

Neither sex nor material nor attention condition had a significant effect on the lateralization difference score in the parietal lobe. They were thus removed from the model.

### PPI results

**FIG. 5.**
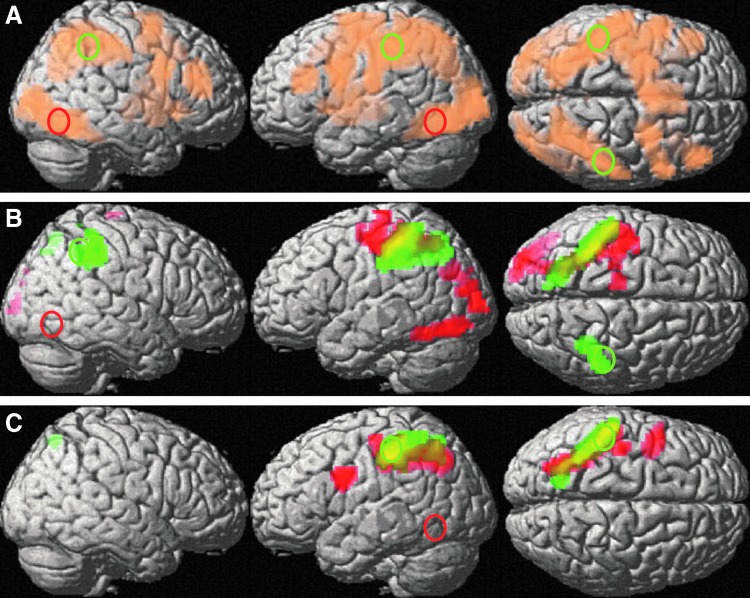
Occipital and parietal connectivity patterns during global–local processing. **(A)** Overall activation pattern of the Navon paradigm (threshold at p_FWE_ < 0.00001). Occipital activation peaks (ROIs) are indicated by red circles, parietal activation peaks by green circles. **(B)** Overall negative connectivity of the right occipital (red) and parietal (green) ROIs during the Navon paradigm. **(C)** Overall negative connectivity of the left occipital (red) and parietal (green) ROIs during the Navon paradigm. ROIs, regions of interest.

All ROIs displayed significant negative connectivity with the left parietal lobe (Fig. 5 and Table 1). The right occipital cortex furthermore displayed negative connectivity with the left occipital cortex. Thus, we observed interhemispheric negative connectivity for right hemispheric ROIs, but intrahemispheric negative connectivity for left hemispheric ROIs. Patterns of positive connectivity are described in Supplementary Data.

**Table 1.** Brain Areas Displaying Negative Connectivity with the Selected Regions of Interest

			*Coordinates*			*Cluster*		
*ROI*	*Brain area displaying negative connectivity with ROI*	*Side*	*x*	*y*	*z*	*Size*	*T*	*p_FWE_*
occL	Superior parietal cortex, postcentral g.	L	−27	−64	46	444	5.3	0.002
	Precentral g.	L	−45	2	31	116	5.3	0.002
occR	Fusiform g./inf. lateral occipital g.	L	−39	−64	−17	203	5.2	0.003
	Lateral occipital cortex	L	−21	−97	7	145	5	0.005
	Superior parietal cortex, postcentral g.	L	−39	−40	61	403	4.8	0.021
parL	Superior parietal cortex, postcentral g.	L	−42	−37	43	325	5.5	0.001
parR	Superior parietal cortex, postcentral g.	L	−42	−43	58	518	6.3	<0.001
	Superior parietal cortex	R	36	−43	40	156	5.2	0.003

occL, left occipital; occR, right occipital; parL, left parietal; parR, right parietal; g., gyrus; ROI, region of interest.

Connectivity patterns did not differ between global and local targets for any ROI and the effect of level (global vs. local targets) was not modulated by sex, material, or attention condition. Consequently, we did not differentiate between global and local targets in further connectivity analyses. All further analyses are based on the average connectivity values for global and local targets extracted from the left occipital and parietal clusters displaying negative connectivity with the right hemispheric ROIs.

#### Occipital lobe

**FIG. 6.**
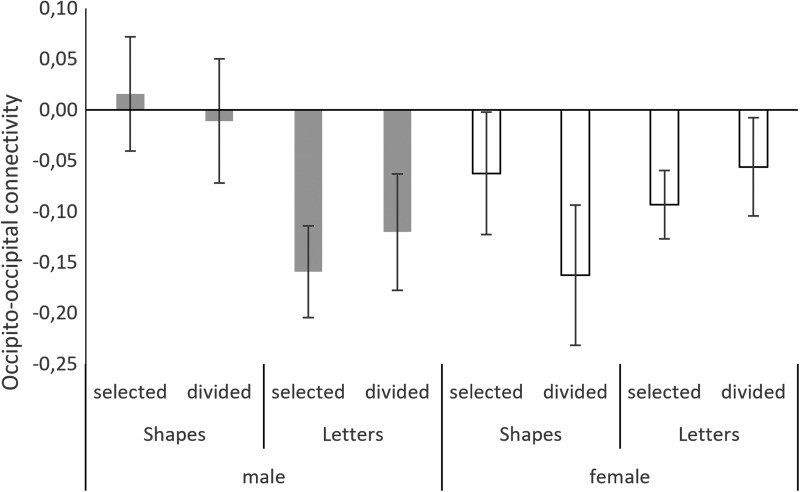
Negative right-to-left interhemispheric connectivity in the occipital lobe. In men, more negative interhemispheric connectivity was observed with letter stimuli than with shape stimuli. In women, negative interhemispheric connectivity was observed in all conditions.

Occipito-occipital connectivity was not affected by attention condition. The main effects of sex (*main effect of sex*: b = −0.31, SE_b_ = 0.14, t_(404)_ = −2.13, *p* = 0.04) and material (*main effect of material*: b = 0.39, SE_b_ = 0.12, t_(404)_ = 3.10, *p* = 0.002) were both significant, as was their interaction (sex*material: b = −0.49, SE_b_ = 0.18, t_(404)_ = −2.78, *p* = 0.006). A stronger negative connectivity was observed for letter stimuli than for shape stimuli in men, but not in women (Fig. 6).

To test for modulatory effects of sex hormones, testosterone and progesterone were entered as predictors in the final model (formula: GA ∼1|PNr + sex*material*hormone). Interhemispheric connectivity in the occipital lobe was not related to sex hormone levels.

#### Parietal lobe

Parietoparietal connectivity was not affected by sex, material, or attention condition and was not modulated by sex hormones.

### Relationship between interhemispheric connectivity and lateralization

To test whether lateralization was related to interhemispheric connectivity and whether this relationship was modulated by sex, material, or attention condition, interhemispheric connectivity values were entered as predictors in the linear mixed models on the lateralization difference scores (formula: LatDiff ∼1|PNr + sex*material*attention*connectivity). To assess the modulatory role of sex hormone levels, testosterone and progesterone values were entered as additional predictors in the final models.

#### Occipital lobe

The lateralization difference score in the occipital lobe was significantly related to occipito-occipital interhemispheric connectivity (*main effect of connectivity:* b = 0.29, SE_b_ = 0.10, t_(171)_ = 2.94, *p* = 0.004). This effect interacted significantly with attention condition (attention*connectivity: b = −0.30, SE_b_ = 0.13, t_(171)_ = −2.33, *p* = 0.02). A more negative interhemispheric connectivity was associated with a smaller lateralization difference during selected attention, but not during divided attention. When sex hormone levels were entered as predictors (formula: LatDiff ∼1|PNr + attention*connectivity*hormone)*,* this association was not modulated by sex hormone levels.

#### Parietal lobe

**FIG. 7.**
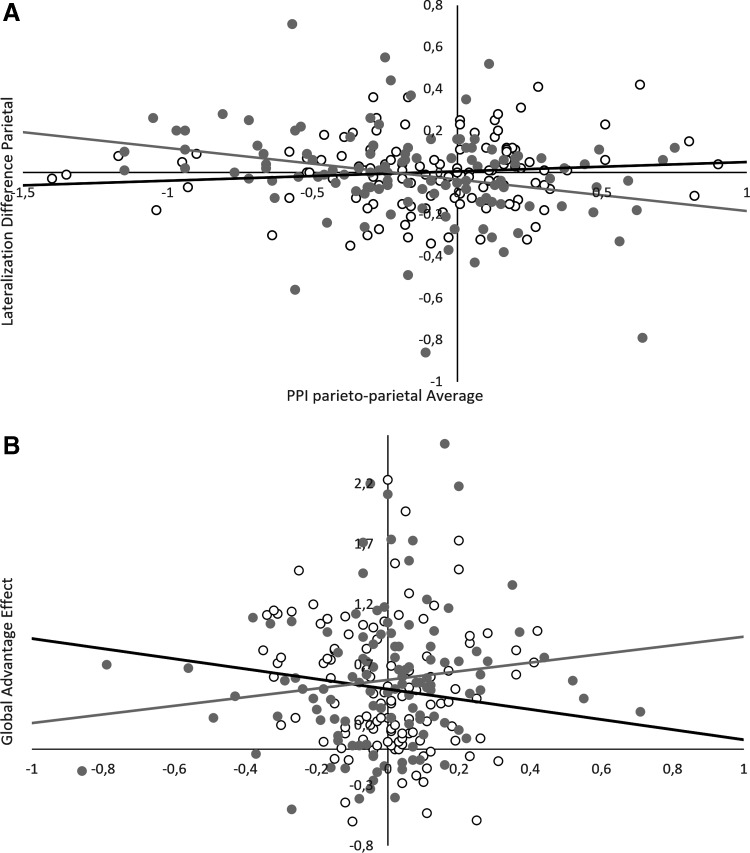
Relationship between negative interhemispheric connectivity, lateralization, and GA in the parietal lobe. In men (gray line), more negative interhemispheric connectivity led to stronger lateralization **(A)**, which, in turn, led to a stronger GA effect **(B)**. In women (black line), the opposite pattern of relationships was observed. Although the association between connectivity and lateralization was mediated through testosterone, the association between lateralization and the GA effect was mediated through progesterone.

The lateralization difference score in the parietal lobe was significantly predicted by parietoparietal interhemispheric connectivity (*main effect of connectivity*: b = −0.50, SE_b_ = 0.13, t_(168)_ = −3.83, *p* < 0.001). This effect interacted significantly with sex and attention condition (sex*connectivity: b = 0.83, SE_b_ = 0.21, t_(168)_ = 4.00, *p* < 0.001, attention*connectivity: b = 0.38, SE_b_ = 0.19, t_(168)_ = 2.12, *p* = 0.04, sex*attention*connectivity: b = −0.73, SE_b_ = 0.27, t_(168)_ = −2.76, *p* = 0.006). In the selective attention condition, a more negative interhemispheric connectivity related to a stronger lateralization difference in men, but weaker lateralization difference in women (Fig. 7a).

When testosterone levels were entered as predictor (formula: LatDiff ∼1|PNr + sex*attention*connectivity*hormone)*,* the effects of sex disappeared and were replaced by effects of testosterone (testosterone*connectivity: b = −0.52, SE_b_ = 0.10, t_(168)_ = −5.40, *p* < 0.001; testosterone*attention*connectivity: b = 0.46, SE_b_ = 0.12, t_(168)_ = 4.01, *p* < 0.001). Progesterone did not modulate the relationship between lateralization and interhemispheric connectivity in the parietal lobe.

### Predicting the GA effect by lateralization and connectivity

To evaluate whether the GA effect was predicted by lateralization or interhemispheric connectivity and whether this association was modulated by sex, material, or attention condition, the lateralization difference scores and interhemispheric connectivity values were entered as predictors in the linear mixed model on the GA effect (formula: GA ∼1|PNr + sex*material*attention*LatDiff*connectivity). To assess the modulatory role of sex hormone levels, testosterone and progesterone values were entered as additional predictors in the final model.

#### Occipital lobe

In the occipital lobe, neither the lateralization difference score (*main effect of LatDiff*: b = 0.003, SE_b_ = 0.06, t_(170)_ = 0.05, *p* = 0.96) nor interhemispheric connectivity *per se* (*main effect of connectivity*: b = −0.02, SE_b_ = 0.06, t_(170)_ = −0.34, *p* = 0.74) did affect the GA effect. However, there was a highly significant interaction between interhemispheric connectivity and the lateralization difference score (LatDiff*connectivity: b = −0.13, SE_b_ = 0.05, t_(170)_ = −2.73, *p* = 0.007). When sex hormone levels were entered as predictors (formula: GA ∼1|PNr + attention+connectivity*LatDiff*hormone), this interaction was not modulated by testosterone or progesterone levels.

#### Parietal lobe

The lateralization difference score in the parietal lobe showed a significant positive association with the GA effect (*main effect of LatDiff*: b = 0.15, SE_b_ = 0.07, t_(170)_ = 2.19, *p* = 0.03), that is, the GA effect was larger, the stronger the lateralization difference between global and local targets in the parietal lobe. A significant interaction with sex (LatDiff*sex: b = −0.31, SE_b_ = 0.12, t_(170)_ = −2.62, *p* = 0.01) indicated that this association was stronger in men than in women (Fig. 7b). Parietoparietal interhemispheric connectivity was related to the GA effect and did not modulate the relationship between parietal lateralization and the GA effect. Therefore, it was removed from the model.

When sex hormone levels were entered as predictors (formula: GA ∼1|PNr + attention+ sex*LatDiff*hormone), the association between parietal lateralization and the GA effect was not modulated by testosterone. When progesterone was entered into the model, the interactive effect with sex disappeared and was replaced by an interactive effect with progesterone (LatDiff*progesterone: b = −0.21, SE_b_ = 0.06, t_(171)_ = −3.30, *p* = 0.001).

**FIG. 8.**
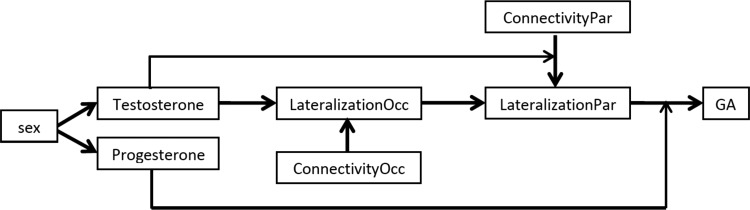
Summary model of the relationships between interhemispheric connectivity, lateralization, and the GA effect. The model is particularly fitting for the selective attention condition. In the occipital lobe, lateralization is higher the lower the participants' testosterone levels and the less negative the interhemispheric connectivity. Parietal lateralization is directly dependent on occipital lateralization but diminished by negative interhemispheric connectivity from the occipital and parietal lobes. The relationship between connectivity and lateralization in the parietal lobe is modulated by testosterone levels, whereas the relationship between lateralization and GA is modulated by progesterone levels.

All observed associations are summarized in Figure 8.

## Discussion

When processing hierarchical stimuli, responses to global targets are faster than responses to local targets (GA effect). Furthermore, global targets are preferentially processed in the right hemisphere, whereas local targets are preferentially processed in the left hemisphere (lateralization). Both the GA effect and lateralization during global–local processing have been related to sex, stimulus material, and attention condition. However, a relationship between the size of the GA effect and the extent of lateralization has not been previously established. Furthermore, lateralization of brain functions has been related to (inhibitory) interhemispheric connectivity. However, interhemispheric connectivity during global–local processing has not been previously investigated. Therefore, this study addresses whether during global–local processing (1) patterns of negative interhemispheric connectivity can be observed and are modulated by sex, material, or attention condition (2) whether this negative interhemispheric connectivity relates to stronger lateralization, and (3) whether the GA effect can be related to said lateralization patterns.

Behaviorally, we observed a larger GA effect with letter stimuli than with shape stimuli and during the selective attention condition than during the divided attention condition. These results are in line with findings of our previous study using the same stimulus material (Pletzer, 2014). Contrary to our previous findings, however, there were no differences in the GA effect between men and women. The most striking difference between our current study and the previous study is the culture/ethnicity of the sample. Although our previous study was performed in southern California and included a large percentage of Asian participants, this study included a Caucasian middle European sample. Previous studies have demonstrated an impact of culture and ethnicity not only on the GA effect but also on interhemispheric connectivity patterns (Mann et al., 1990; Petersson et al., 2007). As in our previous study, however, the GA effect was smaller in participants with higher progesterone levels.

Although sex did not affect the behavioral outcome of global–local processing, it did play an important role in lateralization, interhemispheric connectivity, and their relationship with the GA effect as well as with each other.

Regarding lateralization, we observed the typical global right, local left lateralization in the occipital lobe. This is in line with previous fMRI studies on the Navon paradigm (Fink et al., 1996). Contrary to our expectations, however, this lateralization was stronger in women than in men for the selected attention condition, but irrespective of material or attention condition. First, previous studies suggest a stronger lateralization of brain functions in men than in women (McGlone, 1980; Shaywitz et al., 1995). Note, however, that previous studies have only assessed how strongly whole stimuli are lateralized to the left or right. This is the first study to assess whether men and women differ in how strongly they lateralize different levels of the same stimuli.

Second, previous studies suggest reversed lateralization for shape stimuli (Fink et al., 1997), which was not found in this study. Note, however, that the nature of the shapes utilized in this study was rather abstract and symmetric as compared with the everyday objects utilized by Fink and colleagues (1997). Furthermore, the model of reversed lateralization using object stimuli developed by Fink and colleagues (1997) has also been questioned previously by Hübner and Studer (2009) as well as Bedson and Turnbull (2002).

Third, previous studies suggest stronger lateralization during divided attention conditions (Hübner and Volberg, 2005) than during selective attention conditions. These results are, however, based on EEG studies, which have a much stronger temporal resolution than the fMRI approach used in this study.

Lateralization in the parietal lobe did not differ between global and local targets, although occipital and parietal lateralization were strongly interrelated. The fact that the typical global right and local left lateralization did not extend to the parietal lobes may be explained by the strong pattern of negative intra- and interhemispheric connectivity observed for the left parietal lobe.

A significant pattern of negative right-to-left interhemispheric connectivity was observed for both global and local targets. Specifically, both the right occipital and right parietal lobes were negatively connected to the left parietal lobe. In addition, the right occipital lobe was negatively connected to the left occipital lobe. Interhemispheric connectivity was not affected by attention condition, but was more negative for letter stimuli than for shape stimuli, particularly in men.

Our results as summarized in the model presented in Figure 8 suggest that the relationship between interhemispheric connectivity, lateralization, and behavior is not as straightforward as expected. Specifically, the pattern of relationships cannot be fully explained by the lateralization by inhibition model. First, negative interhemispheric connectivity was observed from the right to the left hemisphere for both global and local targets. Thus, the left hemispheric dominance for local targets does not seem to result from left-to-right interhemispheric inhibition. Rather, the overall negative right-to left interhemispheric connectivity is in line with a general right hemispheric dominance for attention (Heilman and Van Den Abell, 1980: Longo et al., 2015).

Thus, instead of a stronger interhemispheric inhibition leading to a stronger GA effect through lateralization, it appears that the interplay of lateralization and right-to-left interhemispheric connectivity contributes to the behavioral phenomenon. If the right hemisphere inhibits the left during all types of stimuli and local targets are processed in the left hemisphere, responses to the latter may be delayed. Accordingly, neither occipito-occipital interhemispheric connectivity nor occipital lateralization were able to explain the GA effect alone, but their interaction did.

Also, although occipito-occipital connectivity was not affected by attention condition, its relationship with occipital lateralization was, which may, in turn, explain the effect of attention on the GA effect. A more negative interhemispheric connectivity was associated with a smaller lateralization difference during selected attention, that is, the stronger the inhibition of the left hemisphere, the less pronounced was the left hemispheric dominance for local targets in the selected attention condition.

Second, the proposed chain of relationships, i.e. that a stronger interhemispheric inhibition leads to stronger lateralization, which in turn leads to a stronger GA effect, was observed in the parietal lobe for men, but not for women, even though parietal lateralization overall did not differ between global and local targets. The stronger the inhibition from right to left, the stronger was the right hemispheric dominance for global targets and the larger was, in turn, the GA effect. In women, however, the opposite pattern of relationships was observed.

As both excitatory and inhibitory influences have been discussed as potential candidates for explaining lateralization (compare van der Knaap and van der Ham, 2011), the differential results between men and women could indicate differential roles of excitatory and inhibitory connections for lateralization in men and women. This idea is in line with previous results of increased intrahemispheric connectivity in men and increased interhemispheric connectivity in women (Ingalhalikar et al., 2014).

In summary, we propose the following processing models for the Navon paradigm (Fig. 8)

In both men and women, processing of stimuli starts in the occipital lobe. But already in the occipital lobe inhibition is exerted from the right to the left hemisphere, thereby slowing the left hemispheric processing of local information. Then, stimuli are fed forward to the parietal lobe, but the left parietal lobe is inhibited from all sides.

In women, processing of stimuli starts out in a lateralized manner in the occipital lobe. When the lateralized information is fed forward to the parietal lobe, the inhibition of the left parietal lobe diminishes the left hemispheric dominance for local stimuli in the parietal lobe. The stronger this inhibition of the left parietal lobe, the more diminished is the lateralization in the parietal lobe. Relatedly, processing of local information is slowed down in the left hemisphere, leading to GA being the larger the smaller the parietal lateralization.

In men, processing of stimuli does not start out in a strongly lateralized manner, that is, hemispheres in men are less specialized for the local or global level of stimuli. Since there was no lateralization to begin with, a stronger inhibition of the left parietal lobe relates to a stronger lateralization in the parietal lobe. A stronger parietal lateralization is thus related to a stronger slowing of information processing in the left hemisphere.

Importantly, the sex differences in occipital lateralization and the relationship between connectivity and lateralization in the parietal lobe were fully mediated by testosterone levels. This is in line with previous findings of testosterone effects on lateralization (e.g., Toga and Thompson, 2003). Higher testosterone levels reduce the left lateralization of local targets and increase the relationship between interhemispheric connectivity and lateralization. Thus, higher testosterone levels favor interhemispheric processing in line with the lateralization by inhibition model.

Vice versa, sex differences in the relationship between lateralization and the GA effect are fully mediated through progesterone levels. The higher the progesterone levels the more negative was the relationship between lateralization and the GA effect. This may explain on one hand the negative relationship between progesterone and the GA effect. On the other hand, the fact that in women, who show a stronger lateralization in the occipital lobe, subsequent parietal lateralization reduces the GA effect, whereas the opposite is true for men, may explain why there was no behavioral sex difference in the GA effect.

In summary, although in this sample of middle European participants men and women do not differ in their behavioral outcome during the Navon paradigm, they differ substantially in the processing pathways that lead to that outcome. These differential processing patterns are mediated through their hormonal status.

One limitation of this study is the fast presentation of stimuli using a fixed interstimulus interval. Although fast presentation and fixed interstimulus intervals were also used by previous neuroimaging studies on the Navon paradigm (Fink et al., 1996, 1997; Martinez et al., 1997), jittered interstimulus intervals would have resulted in higher power to detect further interrelations between interhemispheric connectivity, lateralization, and behavior beyond those observed in this study.

## Conclusion

In summary, this study suggests that sex hormones modulate the relationship between interhemispheric connectivity, lateralization, and behavior. These results suggest that sex differences in behavior are not so much the result of sex differences in lateralization, but rather lateralization plays differential roles for behavior in men and women. Likewise, sex differences in lateralization are not so much the result of sex differences in interhemispheric connectivity, but rather interhemispheric connectivity plays differential roles for lateralization in men and women.

## Supplementary Material

Supplemental data
