# Supplementary material for: Sex Hormones Modulate the Relationship Between Global Advantage, Lateralization, and Interhemispheric Connectivity in a Navon Paradigm
Source: Brain Connect. 2018 Mar 1;8(2):106–18. doi: 10.1089/brain.2017.0504 (PMC5865260; doi:10.1089/brain.2017.0504)
Supplement: Supplemental data [file Supp_Data.pdf]

Supplementary Data

Patterns of Positive Connectivity

Particularly for the left occipital regions of interest (ROIs), positive connectivity with the deactivation network was stronger in the divided attention condition than in the selected attention condition irrespective of sex or stimulus material (Supplementary Fig. S1A). Furthermore, positive connectivity was stronger in the shapes condition than in the letters condition for all ROIs (Supplementary Fig. 1B). Differential connectivity between letters and shapes was observed in activation areas for occipital ROIs and in deactivation areas for parietal ROIs. Overall connectivity patterns did not differ between men and women, but the difference between shape and letter condition was stronger in women than in men (Supplementary Fig. 1C).

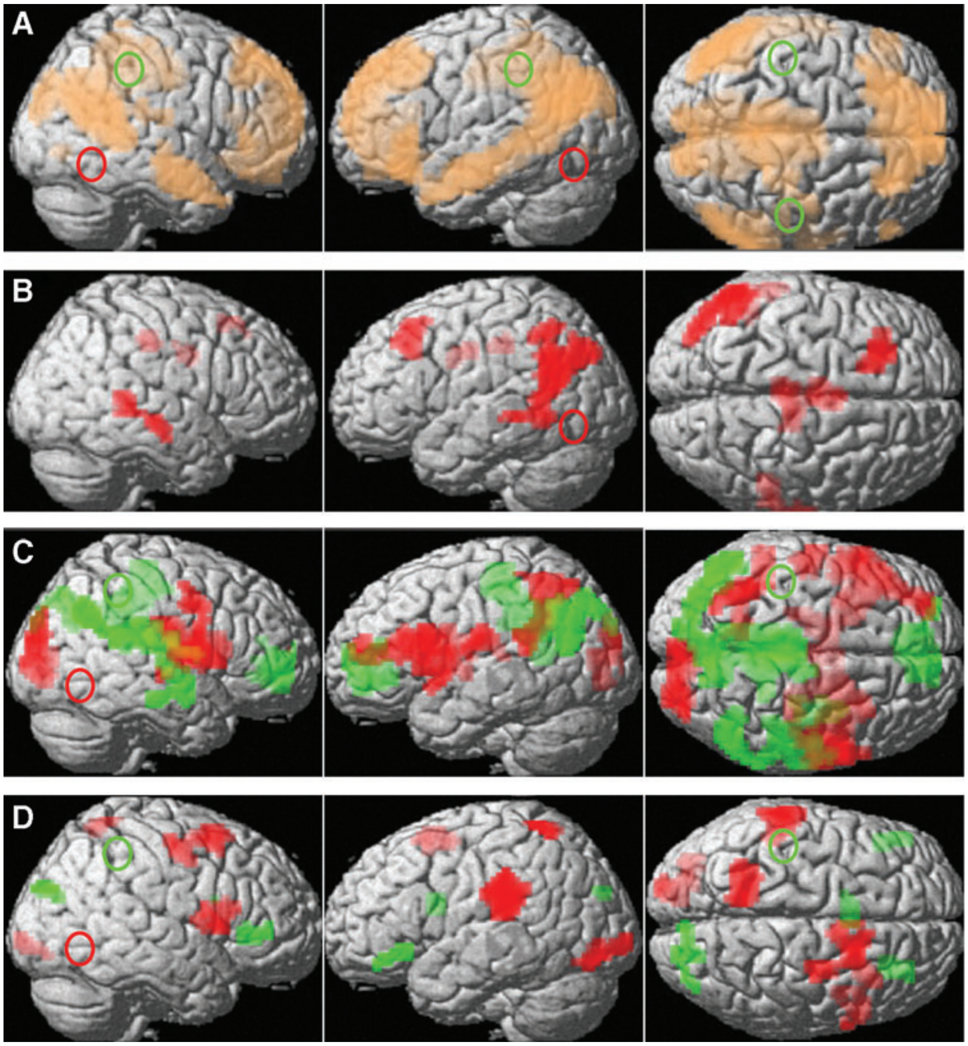

SUPPLEMENTARY FIG. S1. Modulation of connectivity patterns during global–local processing by material and attention condition. **(A)** Overall deactivation pattern of the Navon paradigm (thresholded at $p_{FWE} < 0.00001$). Occipital activation peaks (ROIs) are indicated by red circles, parietal activation peaks by green circles. **(B)** Stronger positive connectivity during divided as opposed to selective attention with the left occipital ROI. **(C)** Stronger positive connectivity during shapes as opposed to letter stimuli with the right occipital (red) and right parietal (green) ROI. **(D)** Areas in which the shape is greater than letter contrast depicted in Panel C is significantly stronger in men than in women. ROIs, regions of interest.

Supplementary Table S1. Occipital and Parietal Activation Peaks in Each Condition

	Left parietal			*Left occipital*			*Right parietal*			*Right occipital*		
Letters divided	−45	−40	46	−45	−70	−11	45	−37	49	48	−58	−11
Letters selected	−42	−40	40	−42	−73	−11	36	−46	46	45	−70	−11
Shapes divided	−42	−40	46	−45	−67	−11	45	−37	46	42	−67	−17
Shapes selected	−45	−37	43	−39	−67	−20	36	−43	43	39	−67	−20
